# The Association between Ambient Fine Particulate Air Pollution and Lung Cancer Incidence: Results from the AHSMOG-2 Study

**DOI:** 10.1289/EHP124

**Published:** 2016-08-12

**Authors:** Lida Gharibvand, David Shavlik, Mark Ghamsary, W. Lawrence Beeson, Samuel Soret, Raymond Knutsen, Synnove F. Knutsen

**Affiliations:** 1 Adventist Health Study-2,; 2 Center for Nutrition, Healthy Lifestyle, and Disease Prevention, and; 3 Center for Community Resilience, School of Public Health, Loma Linda University, Loma Linda, California, USA

## Abstract

**Background:**

There is a positive association between ambient fine particulate matter ≤ 2.5 μm in aerodynamic diameter (PM_2.5_) and incidence and mortality of lung cancer (LC), but few studies have assessed the relationship between ambient PM_2.5_ and LC among never smokers.

**Objectives:**

We assessed the association between PM_2.5_ and risk of LC using the Adventist Health and Smog Study-2 (AHSMOG-2), a cohort of health conscious nonsmokers where 81% have never smoked.

**Methods:**

A total of 80,285 AHSMOG-2 participants were followed for an average of 7.5 years with respect to incident LC identified through linkage with U.S. state cancer registries. Estimates of ambient air pollution levels at participants’ residences were obtained for 2000 and 2001, the years immediately prior to the start of the study.

**Results:**

A total of 250 incident LC cases occurred during 598,927 person-years of follow-up. For each 10-μg/m^3^ increment in PM_2.5_, adjusted hazard ratio (HR) with 95% confidence interval (CI) for LC incidence was 1.43 (95% CI: 1.11, 1.84) in the two-pollutant multivariable model with ozone. Among those who spent > 1 hr/day outdoors or who had lived 5 or more years at their enrollment address, the HR was 1.68 (95% CI: 1.28, 2.22) and 1.54 (95% CI: 1.17, 2.04), respectively.

**Conclusion:**

Increased risk estimates of LC were observed for each 10-μg/m^3^ increment in ambient PM_2.5_ concentration. The estimate was higher among those with longer residence at enrollment address and those who spent > 1 hr/day outdoors.

**Citation:**

Gharibvand L, Shavlik D, Ghamsary M, Beeson WL, Soret S, Knutsen R, Knutsen SF. 2017. The association between ambient fine particulate air pollution and lung cancer incidence: results from the AHSMOG-2 study. Environ Health Perspect 125:378–384; http://dx.doi.org/10.1289/EHP124Citation: Gharibvand L, Shavlik D, Ghamsary M, Beeson WL, Soret S, Knutsen R, Knutsen SF. 2017. The association between ambient fine particulate air pollution and lung cancer incidence: results from the AHSMOG-2 study. Environ Health Perspect 125:378–384; http://dx.doi.org/10.1289/EHP124

## Introduction

Lung cancer (LC) is the leading cause of cancer deaths and the second leading cause of incident cancer among both men and women in the United States with 224,390 new cases and 158,080 deaths expected in 2016 (American Cancer Society 2016). Known risk factors for LC include tobacco smoke (Doll and Hill 1950; Prizment et al. 2014; Weiss 1997), asbestos (Markowitz et al. 2013), arsenic (Chen et al. 2004) and radon (Krewski et al. 2005). According to the International Agency for Research on Cancer (IARC), there is sufficient evidence indicating outdoor air pollution as a cause of LC; the agency has classified outdoor air pollution as well as particulate matter (PM) air pollution, including diesel exhaust (DE), as Group 1 carcinogens (IARC 2013). The findings from several studies, especially the recent results from the European Study of Cohorts for Air Pollution Effects (ESCAPE) (Raaschou-Nielsen et al. 2013), formed the basis for the IARC classification. A meta-analysis by Hamra et al. (2014) reported a positive association between ambient PM and LC incidence and mortality, thus supporting the IARC report. The Diesel Exhaust in Miners Study further elucidated the role of PM since DE is dominated by fine PM. A 5-fold increased estimate of LC was found among miners who had spent significant time using diesel power equipment underground compared to workers who had never worked underground (Attfield et al. 2012).

Given the high fatality rate of LC, studies on mortality and incidence have provided similar results. Studies on the association between LC mortality and ambient fine particulate matter ≤ 2.5 μm in aerodynamic diameter (PM_2.5_) report harmful estimates including a 14% increase in LC mortality in the American Cancer Society (ACS) study (Pope et al. 2002), a 27% increase in LC mortality among women 51–70 years old enrolled in the Oslo Cohort Study (Naess et al. 2007), and a 37% increase in LC mortality in the most versus least polluted cities reported from the Harvard Six Cities Study (Dockery et al. 1993). However, Beelen et al. (2008a) did not find any association with LC mortality in the Dutch Cohort NLCS-AIR Study.

Similarly, for LC incidence, estimates range from 6% to 29% increase with increments of 5–10 μg/m^3^ in PM_2.5_ (Beelen et al. 2008b; Hystad et al. 2013; Puett et al. 2014; Raaschou-Nielsen et al. 2013). When limiting their study population to never and past smokers, the Nurses’ Health Study reported a 37% stronger association with LC for each 10 μg/m^3^ increment in PM_2.5_ (Puett et al. 2014). A new follow-up to the European Study of Cohorts for Air Pollution Effects (ESCAPE) analyzed data from 14 of the cohort studies within the ESCAPE study and reported that the positive association between ambient PM and LC can be attributed to various PM components and sources (Raaschou-Nielsen et al. 2016).

Few studies have assessed the relationship of ozone (O_3_) with LC and most have found no association (Hystad et al. 2013; Vineis et al. 2006). In contrast, in the previous and smaller AHSMOG study, we found an increased LC hazard rate (HR) of 3.56 [95% confidence interval (CI): 1.35, 9.42] for every 100 ppb increment in ambient O_3_ among male study participants (Beeson et al. 1998).

### Objectives

Never-smoking participants have been under-represented in previous cohort studies. The aim of the current study was to assess the association between ambient PM_2.5_ and LC incidence in a health conscious nonsmoking, mostly never-smoking population. Because of our previous findings of an association between ambient O_3_ and LC mortality (Beeson et al. 1998), we also aimed to study the independent relationship with ambient O_3_ in two-pollutant models with PM_2.5_.

## Methods

### Study Population

**Figure 1:**
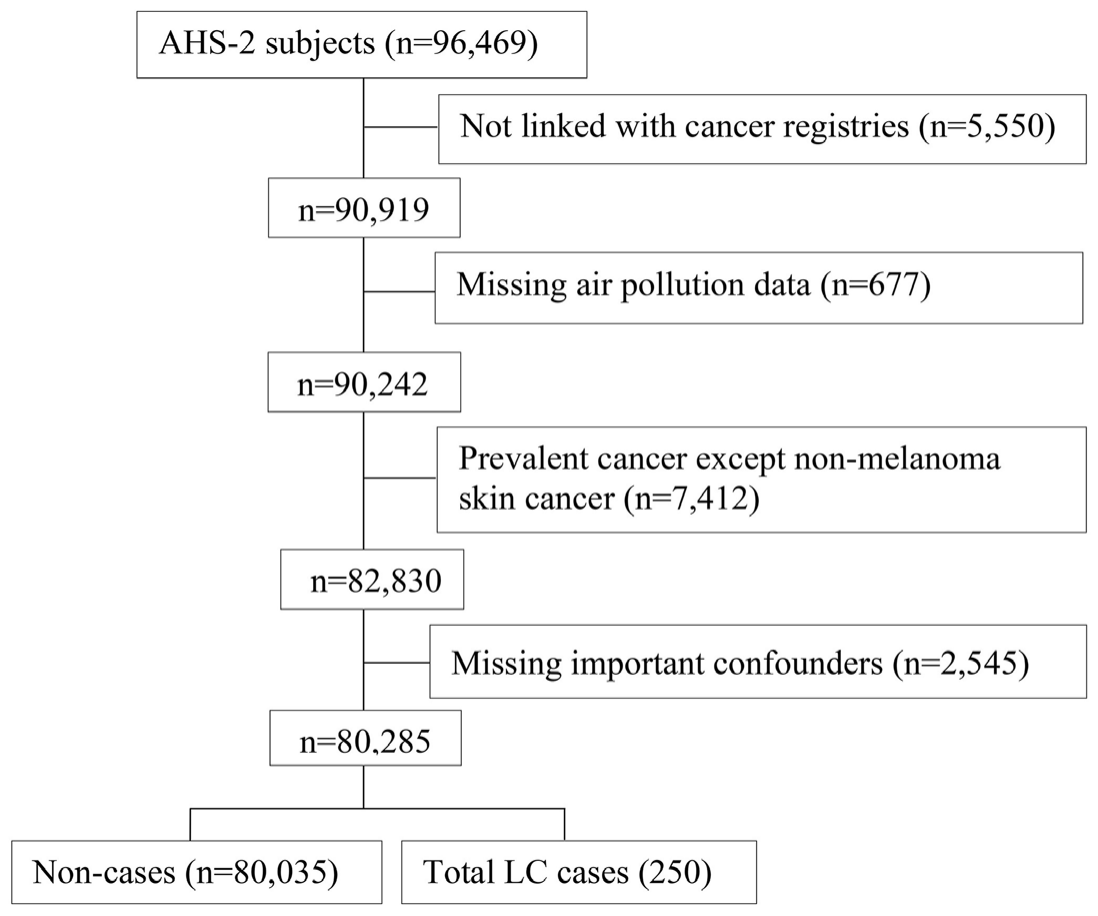
Study flowchart for the final analytic population.

The study population is the AHSMOG-2 study, a large, health conscious cohort of nonsmokers. This is a subpopulation of the Adventist Health Study-2 (AHS-2), a cohort study of about 96,000 participants from all 50 U.S. states as well as 5 provinces of Canada (Butler et al. 2008). Exclusions are shown in Figure 1, which identifies participants not linked with cancer registries (including 4,148 Canadians and 1,402 living in two U.S. states where we were not able to obtain permission to link with the state cancer registry); participants with incomplete address information, which made it impossible to estimate residence-specific air pollution concentrations (*n* = 677); prevalent cancers except non-melanoma skin cancer (*n* = 7,412); missing values on important confounders: age, sex, education levels, hours per day spent outdoors, race, and the nested smoking covariate: smoking status, years since quitting smoking, average number of cigarettes per day (*n* = 2,545).

The final analytic study population consisted of 80,285 participants (Figure 1). Written informed consent was obtained from all participants upon enrollment into the parent study (AHS-2) and this included subsequent analyses using de-identified data. The study was approved by the Loma Linda University Institutional Review Board (IRB) and by the IRBs of participating cancer registries, as required.

### Outcome Assessment

LC cases were identified by ICD-O-3 codes C34.0-C34.9 (WHO 2013) through computer-assisted record linkage of each study participant with state cancer registries (2002–2011). Participants also completed a questionnaire that was mailed biennially regarding newly diagnosed cancers. If such self-reported cancers were not verified through the cancer registry linkage, medical records were obtained to verify such cases (Butler et al. 2008). LC subtypes assessed in this study included squamous cell carcinoma, adenocarcinoma, small cell carcinoma, unspecified carcinoma, and large cell carcinoma. LC cases with histology classification of “other specified” such as lymphoma, carcinoid, and malignant mesothelioma (*n* = 11) were not considered true incident LC and were censored at the time of diagnosis (Figure 1). Thus, the total number of incident LC cases in this study was 250.

### Estimation of Ambient Air Pollution Concentrations

Ambient concentrations of criteria pollutants are measured over a network of hundreds of monitoring stations owned and operated mainly by state environmental agencies. As part of the AHSMOG-2 study, ambient air pollution data were obtained from the U.S. Environmental Protection Agency (EPA) Air Quality System (AQS) for the fixed time period from January 2000 through December 2001: the 2 years immediately prior to the start of the AHSMOG-2 study.

Using the U.S. EPA AQS data and inverse distance weighted (IDW) interpolations methods, monthly pollution surfaces were created for PM_2.5_ and O_3_ across the United States using ArcGIS (ArcMap, version 10.1; ESRI, Redlands, CA). Monthly exposure averages were based on 24-hr O_3_ and daily PM_2.5_ measurements. To minimize errors, the IDW interpolation parameters were selected by assessing the goodness of fit of alternative model configurations through mean prediction error and root-mean-square error estimates. Only months with at least 75% valid data were included in the exposure estimates. The GIS-derived monthly exposure averages were used to accumulate and assign monthly concentrations of ambient O_3_ and PM_2.5_ to the geocoded baseline residential address of the participants.

### Study Covariates

Covariates for the model were selected *a priori* based on published studies and suspected relationships and included sex, race, smoking status, years since participant quit smoking, average number of cigarettes per day during all smoking years, and education level. Additional candidate covariates included calendar time, alcohol consumption, family income, body mass index (BMI), physical activity, and marital status.

In addition, three variables were identified *a priori* as either confounders or effect modifiers: hours per day spent outdoors, years of pre-study residence length at enrollment address, and moving distance from enrollment address during follow-up.

### Statistical Analysis

Baseline characteristics of cases and noncases were compared using chi-square test for categorical and Student’s *t*-test for continuous variables. Cox proportional hazards regression modeling, with attained age as the time variable with left truncation by age at study entry, was used for multivariable analyses. The Cox regression was augmented by adding the sandwich variance estimate (Lin 1994) to adjust for correlated observations within each county. Participants were censored at time of diagnosis or, for noncases, at the time of last linkage with the cancer registry or date of death, whichever came first.

Single- and two-pollutant analyses were conducted. The single-pollutant model assessed the association of ambient PM_2.5_ with LC incidence while the two-pollutant model also included ambient 24-hr O_3_. Pollutants were entered into the model as continuous variables and HRs were calculated for an increment of 10 μg/m^3^ for PM_2.5_ and 10 ppb for average 24-hr O_3_. The increment for PM_2.5_ started with the lowest increment of ambient air pollution registered for this particular cohort.

The multivariable model (Model 1) was specified based on the pollutant(s) and the *a priori* selected covariables. Smoking was used as a nested covariate [i.e., smoke status + (smoke status × years since quit smoking) + (smoke status × years since quit smoking × cigarettes per day)]. We dichotomized years since quitting smoking (< 20 and ≥ 20), and number of cigarettes per day (< 8.5 and ≥ 8.5) based on the median levels. The additional candidate covariates (calendar time, alcohol consumption, family income, BMI, physical activity, and marital status) were evaluated for inclusion in the model, but adding them did not change the main estimate and they were therefore not included in Model 1. Three *a priori* potential effect modifiers (time spent outdoors, residence length, and moving distance during follow-up) were then added to Model 1 as covariates, but this did not change the main association. However, when testing them for multiplicative effect modification, each of them was found to modify the association between PM_2.5_ and LC. Thus, the additional models 3–5 were developed, one for each of these potential effect modifiers. The Cox hazard ratio proportionality assumption was evaluated using Schönfeld residuals, log (−log) plots, and time (attained-age) product terms and no clear departure from proportionality was evident. This was supported further by visual inspection of the log (−log) plots. Furthermore, using multiple linear regressions, no multicollinearity was evident between covariates. Assessment of Schönfeld residuals did not show important influential data points. The linearity assumptions for exposure variables were tested and were not in violation of the proportional assumption.

The following two sensitivity analyses and model checks were performed: *a*) excluding current smokers (*n* = 241) and *b*) excluding unspecified carcinomas of the lung. None of these exclusions changed the main association and therefore they were retained in the *a priori* selected Model 1. A subgroup analysis was also performed to separately assess the estimates of PM_2.5_ in ever and never smokers. All statistical analyses were performed using SAS 9.4 (SAS Institute, Inc. Cary, NC).

## Results

A total of 250 histologically confirmed LC cases (41.7 cases per 100,000 person-years) were diagnosed among the AHSMOG-2 study participants with a median follow-up of 7.5 years (598,927 person-years). Adenocarcinomas constituted 66.4% of all LC (Table 1). Compared to the noncases, cases tended to be women, older, past smokers, with lower educational attainment levels, lower income, and spending more time outdoors. Cases also reported less physical activity, were more likely to have ever consumed alcohol, and had lived longer at their enrollment address. Among cases, ambient PM_2.5_ concentrations were slightly higher (Table 2).

**Table 1:** Incident lung cancers by type, during the 7.5 years of follow-up.

Histology	Total *N* = 80,285	Never smokers		Ever smokers	
		Women *N* = 44,147	Men *N* = 20,759	Women *N* = 8,169	Men *N* = 7,210
Adenocarcinoma	166	65	24	45	32
Squamous cell carcinoma	32	1	4	10	17
Small cell carcinoma	17	4	0	7	6
Large cell carcinoma	2	0	0	1	1
Unspecified carcinoma	33	9	8	5	11
Total LC	250	79	36	68	67
ICD-O-3 histology codes are 8046, 8140, 8250, 8252, 8253, 8255, 8480, 8481, 8550, and 8200 for adenocarcinoma; 8070, 8072, 8074, 8083, and 8560 for squamous cell carcinoma; 8041, 8042, and 8045 for small cell carcinoma; 8012 and 8013 for large cell carcinoma; and 8000, 8010, 8033, 8170, 8720, 8800, 9050, and 9800 for unspecified carcinoma.					

**Table 2:** Selected characteristics of the study population at baseline.

Characteristic	Noncases (*n *= 80,035)	Cases (*n *= 250)	*p*-Value
Age	57.02 ± 14.22	68.75 ± 11.02	< 0.001
Ozone 24 hr (ppb)	26.88 ± 3.89	27.11 ± 4.17	0.344
PM_2.5 _(μg/m^3^)	12.88 ± 3.72	13.18 ± 3.83	0.196
Sex			0.035
Women	52,169 (65.2%)	147 (58.8%)
Men	27,866 (34.8%)	103 (41.2%)
Smoking status			< 0.001
Never smokers	64,791 (81.0%)	115 (46.0%)
Ever smokers	15,244 (19.1%)	135 (54.0%)
Race			0.704
Blacks	22,501 (28.1%)	73 (29.2%)
Nonblacks	57,534 (71.9%)	177 (70.8%)
Education			< 0.001
High school or less	21,888 (27.3%)	124 (49.6%)
Trade school/associate degree/some college	27,186 (34.0%)	78 (31.2%)
Bachelor degree or greater	30,961 (38.7%)	48 (19.2%)
Family income			< 0.001
< $31,000	41,362 (51.7%)	181 (72.4%)
$31,000–$75,000	23,565 (29.4%)	51 (20.4%)
≥ $75,000	15,108 (18.9%)	18 (7.2%)
Body mass index (kg/m^2^)^*a*^			0.213
< 25	30,447 (39.2%)	82 (34.5%)
25–29.99	27,082 (34.9%)	95 (39.9%)
≥ 30	20,060 (25.9%)	61 (25.6%)
Physical activity			0.008
Low	31,474 (39.3%)	121 (48.4%)
Medium	33,520 (41.9%)	95 (38.0%)
High	15,041 (18.8%)	34 (13.6%)
Hours per day spent outdoors			< 0.001
< 1 hr/day	19,545 (24.4%)	49 (19.6%)
1–3.5 hr/day	45,221 (56.5%)	126 (50.4%)
> 3.5 hr/day	15,269 (19.1%)	75 (30.0%)
Alcohol status^*a*^			< 0.001
Never	46,928 (58.9%)	102 (41.1%)
Ever	32,699 (41.1%)	146 (58.9%)
Residence length^*b*^			< 0.001
< 5 years	20,002 (24.9%)	48 (19.2%)
5 ≤ years < 12	20,616 (25.8%)	52 (20.8%)
12 ≤ years < 24	19,755 (24.7%)	61 (24.4%)
≥ 24 years	19,662 (24.6%)	89 (35.6%)
Moving distance^*c*^			0.410
0 km	48,924 (61.1%)	143 (57.2%)
0 < km ≤ 30	15,115 (18.9%)	54 (21.6%)
> 30 km	15,996 (20.0%)	53 (21.2%)
Years since quit smoking (7 levels)			< 0.001
Never smoked	64,791 (81.0%)	115 (46.0%)
Quit ≥ 30 years	4,725 (5.9%)	32 (12.8%)
Quit 20–29.9 years	3,593 (4.5%)	23 (9.2%)
Quit 10–19.9 years	3,155 (3.9%)	32 (12.8%)
Quit 5–9.9 years	1,389 (1.7%)	12 (4.8%)
Quit 1–4.9 years	1,192 (1.5%)	15 (6.0%)
Quit < 1 year or current smokers	1,190 (1.5%)	21 (8.4%)
Average number of cigarettes per day			< 0.001
None	64,791 (80.9%)	115 (46.0%)
Less than average 8.5	7,742 (9.7%)	45 (18.0%)
More or equal than average 8.5	7,502 (9.4%)	90 (36.0%)
Note: Values are presented as mean ± SD or no. (%). ^***a***^Some columns do not add to 100% because of missing data. ^***b***^Years of pre-study residence within 16 km (10 mi) of enrollment address. ^***c***^Distance of moving during follow-up of initial place of residence.			

During follow-up, 20.0% of the participants moved > 30 km from their baseline address, whereas 18.9% moved within 30 km and 61.1% did not change their residence address during follow-up. About 25.0% (20,002 noncases and 48 cases) of cohort participants had lived < 5 years within 16 km (10 mi) of their enrollment address. Thus, their exposure to the ambient air at the enrollment address was relatively short.

Most participants were never smokers (80.8%), 18.9% reported past smoking, of which 54% quit > 20 years ago, and only 0.3% reported current smoking.

**Figure 2:**
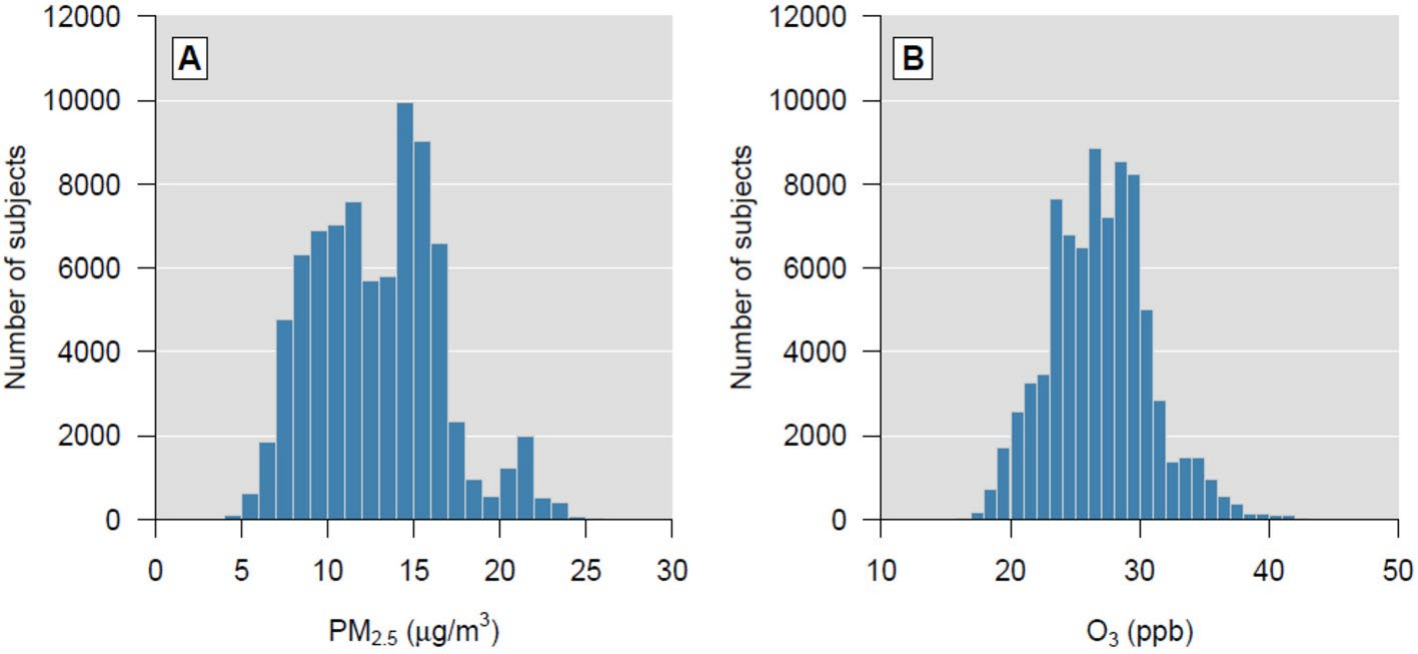
Distribution of the monthly mean concentration of (*A*) PM_2.5_ and (*B*) 24-hr O_3_ (ozone) averaged across the years 2000–2001.

In contrast, among the 250 LC cases, 46.0% were never smokers while 54.0% were past or current smokers (ever smokers) (Table 2). Also, most participants had never used alcohol (58.8%) and only 6.9% were current alcohol users, but with very low intakes. Figure 2A,B shows the distribution of 2-year individual mean ambient concentrations for PM_2.5_ and 24-hr O_3_ for the years 2000–2001. Mean ambient PM_2.5_ concentration was 12.88 μg/m^3^ (range: 4.05–26.55).

### PM_2.5_ and O_3_

A positive association was found between each 10 μg/m^3^ increment in ambient PM_2.5_ and incident LC in both the single-pollutant and two-pollutant sandwich variance estimated model with O_3_ [HR = 1.42 (95% CI: 1.02, 1.98)] and [HR = 1.43 (95% CI: 1.03, 2.00)], respectively (Table 3). Comparable estimates, in the two-pollutant models with O_3_, were observed among ever smokers [HR = 1.49 (95% CI: 1.02, 2.18)] and never smokers [HR = 1.32 (95% CI: 0.90, 1.93)]. A weak association with LC was found for each 10 ppb increment in 24-hr O_3_ [HR = 1.07 (95% CI: 0.78, 1.48)] in the two-pollutant multivariable model (Model 1, Table 3).

**Table 3:** Multivariable-adjusted HRs for incident lung cancer per 10-μg/m^3 ^increment in mean monthly ambient PM_2.5_: single- and two-pollutant models.

Model	Pollutant	Cases	HR (95% CI)		
			Single pollutant	Two pollutant^*a*^	Two pollutant^*a,b*^
Model 1	PM_2.5_	250	1.42 (1.02, 1.98)	1.43 (1.03, 2.00)	1.43 (1.11, 1.84)
	O_3_			1.07 (0.78, 1.48)	1.07 (0.78, 1.47)
Model 2	PM_2.5_	250	1.45 (1.04, 2.03)	1.46 (1.05, 2.05)	1.46 (1.13, 1.89)
	O_3_			1.08 (0.78, 1.49)	1.08 (0.79, 1.47)
Model 3
Outdoors < 1 hr/day	PM_2.5_	49	0.76 (0.36, 1.63)	0.77 (0.36, 1.64)	0.77 (0.42, 1.42)
Outdoors ≥ 1 hr/day	PM_2.5_	201	1.67 (1.16, 2.42)	1.68 (1.17, 2.44)	1.68 (1.28, 2.22)
Model 4
Residence < 5 years	PM_2.5_	48	1.06 (0.51, 2.19)	1.06 (0.51, 2.20)	1.06 (0.46, 2.48)
Residence ≥ 5 years	PM_2.5_	202	1.53 (1.06, 2.21)	1.54 (1.07, 2.24)	1.54 (1.17, 2.04)
Model 5
Distance ≤ 30 km	PM_2.5_	197	1.37 (0.94, 2.00)	1.38 (0.95, 2.02)	1.38 (1.04, 1.83)
Distance > 30 km	PM_2.5_	53	1.66 (0.84, 3.26)	1.68 (0.85, 3.31)	1.68 (0.94, 2.98)
Note: Models based on data of 80,285 AHSMOG-2 participants (LC cases: *n* = 250). ^***a***^Model (1–5)–adjusted for O_3_ (ozone) with increments of 10 ppb. ^***b***^Model (1–5)–with Sandwich variance estimate. Model 1–Adjusted for sex, education level, race, and nested covariates: smoking status, years since quitting smoking, and average number of cigarettes per day. Model 2–Model 1 + outdoors, residence length, moving distance. Model 3–Model 1 + outdoors + PM_2.5_ × outdoors (2 levels of outdoors: < 1 and ≥ 1 hr/day). Model 4–Model 1 + residence + PM_2.5_ × residence (2 levels of residence: < 5 and ≥ 5 years). Model 5–Model 1 + distance + PM_2.5_ × distance (2 levels of distance: ≤ 30 and > 30 km).					

### Effect Modifications

The three *a priori* identified potential effect modifiers (time spent outdoors, residence length, and moving distance) were found to modify the association between PM_2.5_ and LC (models 3–5) (Table 3). For time spent outdoors, there was no association between PM_2.5_ and LC among those spending < 1 hr/day outdoors. However, for those spending > 1 hr/day outdoors, there was a 68% increase in the estimate for LC [HR = 1.68 (95% CI: 1.28, 2.22)] (Table 3). Similarly for those who had lived < 5 years within 10 mi (16 km) of their enrollment address, there was no association between ambient PM_2.5_ and LC. However, among those having lived > 5 years at or close to their enrollment address, the estimate for incident LC increased to 54% [HR = 1.54 (95% CI: 1.17, 2.04)]. For those who had moved > 30 km during follow-up, the estimate was somewhat higher [HR = 1.68 (95% CI: 0.94, 2.98)] compared to those who had not moved or moved < 30 km from their enrollment address [HR = 1.38 (95% CI: 1.04, 1.83)].

### Sensitivity and Subgroup Analyses

When we excluded the very small group of current smokers (2 cases of LC among 241 current smokers), the HR remained unchanged. When we excluded 33 cases with unspecified carcinoma of the lung, the HR became slightly stronger at 1.45 (95% CI: 1.10, 1.92). Finally, when comparing never and ever smokers, the HRs associated with each 10 μg/m^3^ were comparable at 1.32 (95% CI: 0.90, 1.93) and 1.49 (95% CI: 1.02, 2.18), respectively.

## Discussion

Not surprisingly, the majority of the LC cases in this study (66.4%) were adenocarcinomas, given that virtually all participants were nonsmokers. The Nurses’ Health Study found a similar proportion with 51% of LC being adenocarcinomas among never smokers or those who quit smoking ≥ 10 years ago (Puett et al. 2014). The overall LC incidence rate was 41.7 per 100,000 person-years in this cohort, compared to 78.6 for men and 54.6 for women in the general U.S. population (2007 to 2011) (Siegel et al. 2015).

Three of the four studies on ambient PM_2.5_ concentrations and LC incidence reported positive HRs ranging from 1.06 (95% CI: 0.91, 1.25) to 1.29 (95% CI: 0.95, 1.76) for each 10 μg/m^3^ increment in ambient concentrations of PM_2.5_ (Hystad et al. 2013; Puett et al. 2014; Raaschou-Nielsen et al. 2013). The Netherlands Cohort Study on Diet and Cancer, however, did not find any association with PM_2.5_ [HR = 0.81 (95% CI: 0.63, 1.04)] (Beelen et al. 2008b). A recent meta-analysis of the relationship between PM_2.5_ and LC incidence and mortality reported a meta-relative risk (RR) of 1.09 (95% CI: 1.04, 1.14) for the full meta-estimate of all studies included in the meta-analysis, and RR = 1.18 (95% CI: 1.00, 1.39) for never smokers, for each 10 μg/m^3^ increment in ambient concentrations of PM_2.5_ (Hamra et al. 2014). Also, in a Canadian cancer registry-based case–control study using LC cases accrued between 1975–1994, and spatio-temporal models for assessment of ambient air pollution, a 29% [OR = 1.29 (95% CI: 0.95, 1.76)] increase in LC incidence was reported with each 10-μg/m^3^ increment in PM_2.5_ and 9% [OR = 1.09 (95% CI: 0.85, 1.39)] increase for each 10 ppb increase in O_3_ (Hystad et al. 2013). The results of the present study are in agreement with the weight of prior evidence and the recent determinations by the IARC Working Group classifying outdoor air pollution and particulate matter as carcinogenic (Group 1) (IARC 2013). Depending on the model, our HR estimates range from 1.43 (95% CI: 1.11, 1.84) to 1.68 (95% CI: 1.17, 2.44) per 10 μg/m^3^ increment in PM_2.5_ and this is higher than the other studies on LC incidence.

Smoking seems to modify the association of ambient air pollution with LC incidence. The Nurses’ Health Study, in a follow-up from 1994 through 2010, found a positive, but weak, association with incident LC with HR = 1.06 (95% CI: 0.91, 1.25) for each 10-μg/m^3^ increment in PM_2.5_. However, the HR was 1.37 (95% CI: 1.06, 1.77) and closer to our findings when limiting analyses to never smokers and those who had quit smoking ≥ 10 years ago (Puett et al. 2014). The Netherlands Cohort Study on Diet and Cancer did not find an association between LC and ambient PM_2.5_ levels. It is unclear why the Netherlands Cohort Study on Diet and Cancer reported null findings, but it could possibly be due to the high prevalence of current and past smokers, which would be in line with the weak findings in the Nurses’ Health Study before smokers were excluded. However, the Netherlands Cohort Study on Diet and Cancer reported stronger associations between black smoke exposure estimates and incident LC among never smokers as compared to former and current smokers with HR = 1.47 (95% CI: 1.01, 2.16), HR = 0.91 (0.68, 1.23) and HR = 0.85 (95% CI: 0.70, 1.03), respectively (Beelen et al. 2008b). Hystad on the other hand, found stronger associations of PM_2.5_ among former [HR = 1.45 (95% CI: 0.96, 2.19)] and current smokers [HR = 1.17 (95% CI: 0.75, 1.84)] than among never smokers [HR = 0.95 (0.38–2.34)] (Hystad et al. 2013). In our study, the association between PM_2.5_ and LC incidence among former and never smokers was comparable, although slightly stronger among former smokers, HR = 1.49 (95% CI: 1.02, 2.18) and HR = 1.32 (95% CI: 0.90, 1.93), respectively. The similar estimates probably reflect the fact that our past smokers had quit smoking on average 24 years ago and thus there is less residual confounding by smoking.

The present study has assessed possible effect modification of time spent outdoors on the association between ambient air pollution and incident LC. Besides the strength of studying a nonsmoking and mostly never-smoking population, our ability to include effect modification by both time spent outdoors and length of residence at enrollment address can possibly explain our stronger findings. When limiting our analyses to those who had lived within 10 mi of their enrollment address for > 5 years, our estimates increased substantially from HR = 1.43 (95% CI: 1.11, 1.84) to HR = 1.54 (95% CI: 1.17, 2.04) (Table 3). This is in line with the Nurses’ Health Study that also found that the HR increased when limiting their study population to those who had not moved between 1976 and 1994, the years immediately prior to the start of the LC follow-up from 1994 through 2007 (Puett et al. 2014). Given the long latency period for cancers, this result would be expected. Similarly, the Danish study reported an increase in HR of total LC incidence from HR = 1.18 (95% CI: 0.96, 1.46) to HR = 1.33 (95% CI: 0.98, 1.80) when excluding those who had moved during the 12.8 years follow-up (Raaschou-Nielsen et al. 2013). In our study, however, such an association was less clear, possibly due to our relatively short follow-up and the long latency time for LC.

**Figure 3:**
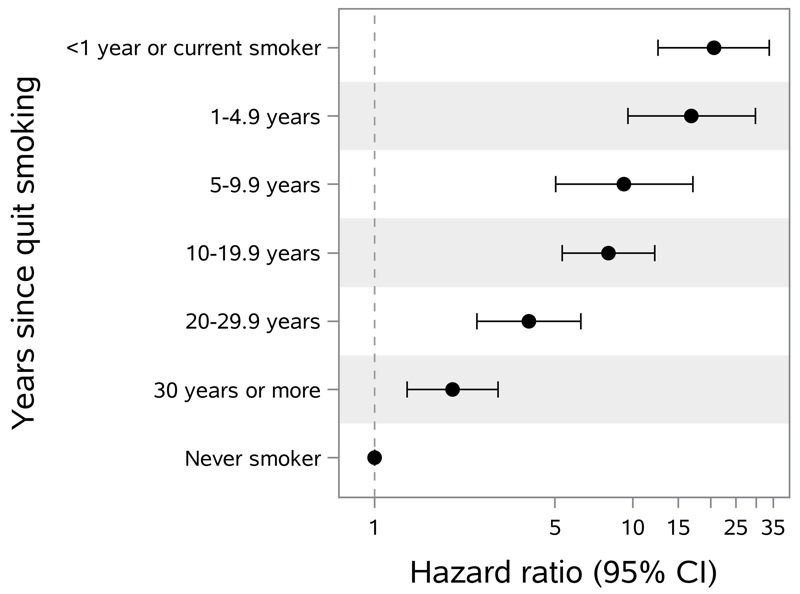
Hazard ratios of incident lung cancer in the study population stratified by time since quitting smoking among ever smokers (135 cases) compared to never smokers (115 cases).

Our study participants are health conscious, mostly nonsmokers, about 50% adhere to plant-based diets, and engage in medium to high physical activity. Nonetheless, we found similar associations of known risk factors for LC as other studies have reported. Specifically, we found that HR of incident LC decreased with increasing number of years since study participants quit smoking (Figure 3). A similar monotonic association has been reported with increments of cigarettes per day in the ACS Cancer Prevention Study II (Pope et al. 2011).

### Biologic Mechanisms

DNA damage and cell cycle alterations are among the biological mechanisms that have been suggested to explain the association between PM_2.5_ and LC (Longhin et al. 2013; Sørensen et al. 2005). Exposing human bronchial epithelial cells *in vitro* to PM_2.5_, Longhin et al. (2013) observed increased DNA damage that resulted in severe mitotic spindle defects and elevated number of cells having micronuclei, measures that have been reported in other investigations to have a strong correlation with the risk of LC (El-Zein et al. 2008; McHugh et al. 2013). Additionally, PM_2.5_ was also associated with elevated production of reactive oxygen species (Longhin et al. 2013), which previously has been reported to increase cancer risk through oxidative DNA damage, impairment of oncogene suppressor genes and induction of malignancy transformation (Waris and Ahsan 2006). Furthermore, a previous investigation reported that analyzed blood lymphocytes and 24-hr urine samples of participants exposed to PM_2.5_ to assess the role of PM_2.5_ in oxidative stress found that transition metals contained in PM_2.5_, including vanadium and chromium, were responsible for oxidative DNA damage that were independent of other compounds in the mixture (Sørensen et al. 2005). To summarize, it appears that PM_2.5_ causes cell cycle alterations and DNA damage mainly through the production of reactive oxygen species that are inhibited by the presence of antioxidants (Longhin et al. 2013).

### Strengths and Limitations of the Study

There are several strengths of this study. The target population is health conscious, and the use of tobacco is very low. This nonsmoking, mostly never smoking, population boosts power to evaluate the association between ambient air pollution and incident LC in the absence of confounding by current or former smoking. Another strength is that this is a population living across the United States in both urban and rural communities. Because this population seems to reside in areas with relatively low concentrations of ambient PM_2.5_, it provides a unique opportunity to study possible health effects of ambient PM_2.5_ even at relatively low concentrations. The fact that we were able to assess the effect modification of time spent outdoors, length of residence at enrollment address, and moving history during follow-up are strengths that add to our understanding of the role of these variables when assessing the association between ambient air pollution and LC.

We did not have specific information on environmental tobacco smoke (ETS) in our data and this is a potential limitation. However, we believe the prevalence of ETS is very low in this population given the fact that most Adventists live in nonsmoking households with other Adventists. Also, there was no information on how many hours the participants spent traveling in motor vehicles to and from work that would expose them to traffic air pollution, which is known to have higher concentrations of PM_2.5_ than typical residential areas (Brown et al. 2012; Knibbs et al. 2010; Mirabelli et al. 2015; Weichenthal et al. 2015). Such information at the individual level could potentially modify the observed associations we have reported. Additionally, residence-specific air pollution estimates were based on air quality monitoring stations and this may result in unknown amounts of misclassification. However, such misclassification is likely to be nondifferential and would thus tend to bias results towards the null. Finally, our data lacked any information regarding the speciation and components of PM_2.5._ In spite of the recent paper from the ESCAPE study (2016), it is still unclear whether the particle size per se or the chemicals coating the particles are the culprit for the observed association with LC. Further studies on the individual effects of various components of PM_2.5_ are needed to better understand the association between air pollution and development of LC.

## Conclusions

In summary, this study found increased estimates of incident LC associated with each 10 μg/m^3^ increment of ambient PM_2.5_ in a study population consisting mainly of never smokers who lived in areas with relatively low concentrations of ambient PM_2.5_. The observed relationship was in line with, or somewhat stronger than, what has been reported by most other studies and was independent of both active smoking and ambient O_3_ concentrations. There was no independent association between incident LC and ambient 24-hr O_3_ concentrations. The association between ambient PM_2.5_ and incident LC was comparable among ever and never smokers.

The results of the present study support the conclusions of the IARC in classifying outdoor air pollution and PM as carcinogenic. Furthermore, our findings of substantial positive associations between incident LC and PM_2.5_, even at relatively low ambient concentrations, have important public health implications, especially for never and past smokers, in regards to making informed decisions on place of residence. Also, our findings could have implication for national ambient air quality standards for PM_2.5_ established by the U.S. Environmental Protection Agency.
